# Comparative Effectiveness of Treatments for Bacterial Vaginosis: A Network Meta-Analysis

**DOI:** 10.3390/antibiotics10080978

**Published:** 2021-08-13

**Authors:** Alison Muñoz-Barreno, Fausto Cabezas-Mera, Eduardo Tejera, António Machado

**Affiliations:** 1 Instituto de Microbiología, Colegio de Ciencias Biológicas y Ambientales (COCIBA), Campus Cumbayá, Universidad San Francisco de Quito (USFQ), Diego de Robles y Vía Interoceánica, Quito 170901, Ecuador; amunozb@estud.usfq.edu.ec (A.M.-B.); fscabezasm@estud.usfq.edu.ec (F.C.-M.); 2 Grupo de Bioquimioinformática, Facultad de Ingeniería y Ciencias Agropecuarias Aplicadas, Universidad de Las Américas, Quito 170125, Ecuador

**Keywords:** bacterial vaginosis, antibiotics, probiotics, combined therapies, randomized controlled trials, meta-analysis

## Abstract

Bacterial vaginosis (BV) is a common vaginal dysbiosis in women of reproductive age. However, the cure rate for BV varies considerably and many women experience a relapse after the initial treatment. The present meta-analysis aimed to evaluate the clinical cure rates (CCRs) in randomized controlled trials (RCTs) through different therapies and administration routes. This meta-analysis included a final set of 25 eligible studies with a total of 57 RCTs and compared the effectiveness of BV treatments among non-pregnant and pregnant women. The initial range of CCRs varied greatly from 46.75% to 96.20% and the final pooled CCR was 75.5% (CI: 69.4–80.8) using the random model. The heterogeneity indices were Q = 418.91, I2 = 94.27%, and τ = 0.7498 (*p* < 0.0001). No publication bias was observed according to Funnel plot symmetry and Egger’s linear regression test (*p* = 0.1097). To evaluate different variables, sub-group analysis, meta-regressions, and network meta-analysis were also realized. The highest P-scores in CCR were obtained by: (1) a combined therapy with local probiotic treatment and application of antibiotics by both administration route (oral clindamycin and local 5-nitroimidazole; P-score = 0.92); (2) a combined therapy with oral administration of 5-nitroimidazole and probiotic treatment (P-score = 0.82); (3) and a combined therapy with local administration of 5-nitroimidazole and oral probiotic treatment (P-score = 0.68). A clear-cut decision of the best BV treatment was not possible due to the heterogeneity of outcomes reported in the trials, indicating the necessity for a better characterization of RCTs. Finally, combined therapies suggested the reduction of the optimal concentration of antibiotics, and double phase treatments of antibiotics indicated an increment of CCRs in BV.

## 1. Introduction

Bacterial vaginosis (BV) is a common vaginal microbiota dysbiosis in women of reproductive age. The prevalence of BV in the United States is 29%, while in Europe it is 4–14% [1]. BV is a dysbiosis characterized by a reduction of *Lactobacillus* species, such as *L. crispatus, L. gasseri,* and *L. jensenii*, being replaced by various anaerobic bacteria, which includes *Gardnerella vaginalis*, *Mycoplasma hominis*, *Atopobium vaginae*, *Peptostreptococcus* sp., *Prevotella* sp., and *Mobiluncus* species [1,2,3]. BV may occur along with other vaginal infections (as vaginal candidiasis or trichomoniasis), increasing the risk of acquiring sexually transmitted infections (STIs) and pre-term births [1,4]. Although the etiology has not been fully understood, many factors may promote this dysbiosis development, such as age, pregnancy, sexual intercourse, and the use of antibiotics or contraceptives [5]. Despite epidemiological studies revealed that genetic host immunity, ethnicity, and vaginal microbiota among women differed and BV treatment need to be adjusted [4,6,7,8,9], most randomized controlled trials only evaluated different types of treatments. However, pregnant women are described to be more susceptible to acquire BV and more vulnerable to relapse after initial treatment [10]. In addition, standard BV treatment can vary both within and between countries after its diagnosis in women, reporting different clinical cure rates [11].

There are different diagnostic methods to diagnose BV, such as Amsel Criteria and Nugent Score [12]. Although Nugent Score had previously been considered to be the gold standard for BV diagnosis, the Amsel criteria have replaced it in the clinical diagnostic method providing a more accessible and clinically defined basis for the diagnosis through only four criteria. Three of the four criteria must be present to confirm the diagnosis, those being: (1) thin, white, yellow, homogeneous discharge; (2) Clue cells on wet mount microscopy; (3) a vaginal fluid pH of over 4.5 when placing the discharge on litmus paper; and, (4) the release of a fishy odor when adding 10% potassium hydroxide (KOH) solution to wet mount, also known as a “whiff test” [13]. Therefore, most randomized controlled trials (RCTs) evaluate the clinical cure rates (CCRs) in women using Amsel Criteria. The present study retrieved all RCTs that evaluated BV treatments using Amsel Criteria as diagnostic method. 

Treatment for this dysbiosis generally involves antibiotic therapy via intra-vaginal gel or oral pill, being metronidazole or clindamycin the most common anti-microbial drugs. However, the cure rate for BV may vary between 65 to 85%, and many women experience a relapse weeks or months after the initial treatment [14]. Moreover, reports on anti-microbial resistance of BV pathogens and low long-term cure rates have been increasing in the last years [15,16,17]. Simultaneously, probiotics have been proposed as an alternative treatment for BV applying live micro-organisms with the capacity to confer health benefits to the patient. Lactobacilli are the probiotics most often used to treat BV [17]. Several studies reported positive results in clinical trials, supporting the use of lactobacilli as an alternative or even as conjugate treatment together with antibiotics to increase the CCRs [18,19]. Although other non-lactobacilli microorganisms are also known by their probiotic activity [6], most RCTs only analyzed *Lactobacillus* species in probiotic BV treatments. 

The present study aimed to evaluate the effectiveness of treatments for BV after an initial therapy on women, analyzing the efficiency and significant differences between therapies and administration routes. Therefore, we assessed the CCRs of different clinical treatments based on antibiotics, probiotics, and conjugates, as well as routes of administration from published studies around the world. This study attempted to obtain a general picture of the effectiveness and trends among BV treatments in pregnant and non-pregnant women through meta-analysis.

## 2. Results

### 2.1. Study Inclusion Criteria and Characteristics of the Eligible Studies

**Figure 1:**
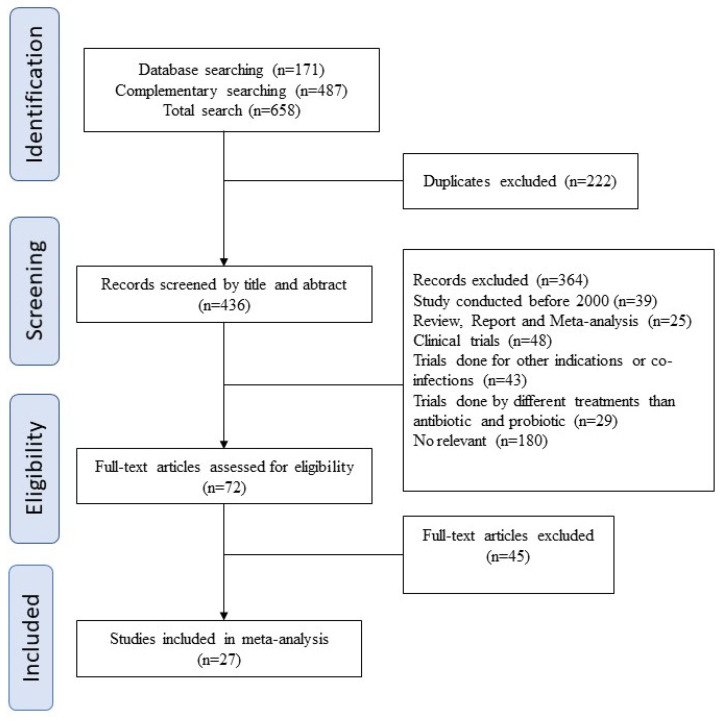
Prisma flow chart of the eligible studies obtained during the screening process.

A total of 658 studies were retrieved and 72 full texts were reviewed. Twenty-nine studies met our inclusion criteria. The final data set include studies covering different global regions (most of them in Europe). All available and relevant data were extracted from each study (more exactly, type of treatment, route of administration, clinical cure rate, reinfection rate, and pregnant or non-pregnant state). These data were then used to create another file base, selecting only information reported in five or more papers, and consequently, each paper was cited more than once (see Figure 1).

A total data set of 27 studies were obtained for the present meta-analysis following the eligibility criteria, screening process, and quality assessment, being further processed to evaluate CCR reports.

### 2.2. The Overall Efficiency of Bacterial Vaginosis Treatments

The data set reported CCR of bacterial vaginosis treatments between 2000 and 2018 in several countries worldwide. As shown in Table 1, the values of CCR varied greatly from 46.75% to 96.20% among eligible studies. Different types of treatment were also described, evaluating the exclusive therapy by antibiotics (AB: 23/27) or probiotics (PB: 6/27), and even combined therapies (AB + PB: 11/27).

Most of the data set belonged to studies realized in Europe (14/27), followed by Asia (5/27), America (4/27), Africa (3/27), and finally Oceania (1/27). However, three-fourths of the studies in America belonged to the United States of America (USA), and just one study was from Brazil. Likewise, three-fifths of the studies in Asia belonged to India, and two-thirds of the studies in Africa were from Nigeria. Finally, four studies in our data set reported the CCR of bacterial vaginosis treatments among pregnant women.

**Figure 2:**
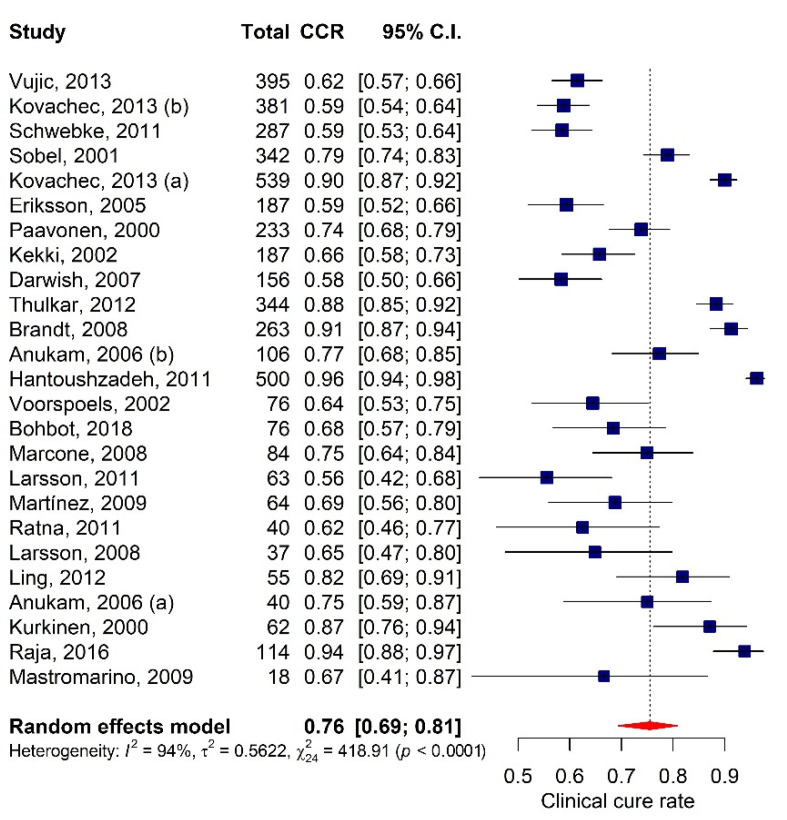
Forest plot of the meta-analysis of CCR of treatments for bacterial vaginosis.

After removing the two outliers of the initial data set of 27 studies [31,40], the final pooled clinical cure rate was 75.5% (CI: 69.4–80.8) and the heterogeneity indices computed using the random model were: Q = 418.91, I2 = 94.27%, and τ = 0.7498 (*p* < 0.0001), as shown in Figure 2. The final set of 25 eligible studies reported a total of 57 RCTs, comparing the effectiveness of BV treatments with different doses of antibiotics and/or probiotics through oral and local administration (for further information see the General screening database in Supplementary file).

**Figure 3:**
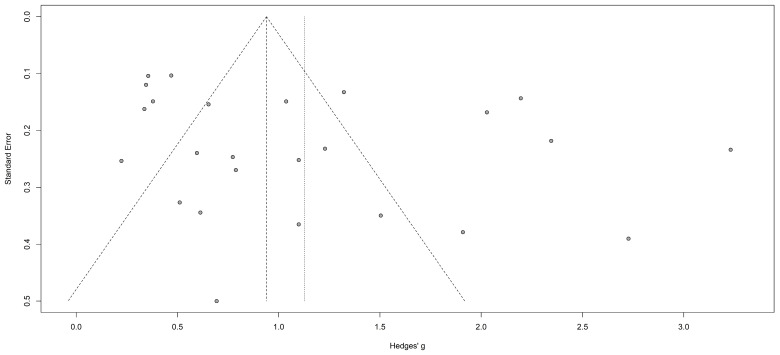
Funnel plot of the meta-analysis on the clinical cure rate of treatments for bacterial vaginosis. An Egger test was used to detect asymmetry in the funnel plot that could suggest the presence of publication bias (*p* = 0.1097).

A funnel plot was then realized to evaluate the existence of publication bias in the final data set (see Figure 3). Egger’s linear regression test was also used to reveal any publication bias and possible asymmetric data distribution in the selected studies. No publication bias was observed according to Funnel plot symmetry and Egger’s linear regression test (*p* = 0.1097).

### 2.3. P-Curve Analysis and Detection of P-Hacking 

**Figure 4:**
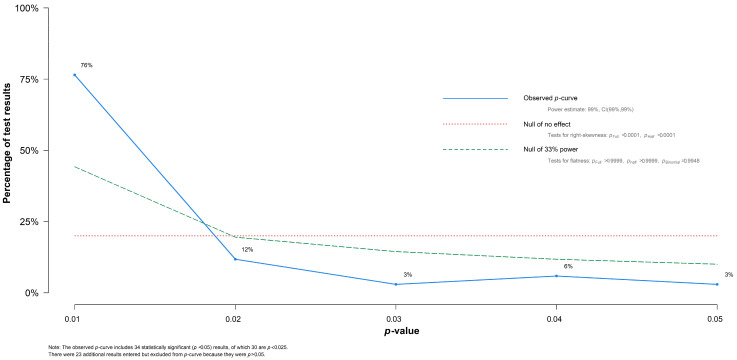
*P*-curve was used to assess publication bias and detect *p*-Hacking.

To evaluate different variables in the effectiveness of BV treatment, sub-group analysis, meta-regressions, and network meta-analysis were realized among our data set. However, the presence of publication bias could lead to data mining, and so an evaluation of *p*-curve was realized. As shown in Figure 4, the *p*-curve analysis supports the absence of publication bias in our overall and sub-group results. The detection of *p*-Hacking allowed us to observe the distribution of statistically significant *p*-values in our data set.

The *p*-curve analysis illustrated a shape with several evidential values of the test results. From our initial 57 test results included into the sub-group analyses and meta-regressions, 34 test results were statistically significant values (*p* < 0.050), and of those, 30 test results possessed a *p*-value lower than 0.025 and the curve generated was right-skewed, suggesting a set of significant *p*-values among our eligible studies.

### 2.4. Effectiveness of BV Treatment Types, Administration Routes, and Pregnancy State

The effectiveness of BV treatment between antibiotics, probiotics, and conjugate or combined therapies was evaluated through sub-group analysis. As shown in Table 2, 57 randomized controlled trials (RCTs) were considered from our final data set for the evaluation of the CCR among different treatment types. Although the CCRs of probiotic therapy overpassed the effectiveness of both antibiotic and conjugate or combined therapies, no statistically significant difference was obtained among the pooled CCR between treatment types (*p* = 0.845).

No publication bias was found in the evaluated sub-groups according to Egger’s linear regression test among conjugate or combined therapy. However, it was not possible to apply Egger’s linear regression test in probiotic therapy due to the low number of trials (k ≤ 10). In addition, antibiotic therapy showed a low *p*-value (*p* = 0.091) compiling CCRs of 35 trials, where it was possible to detect some heterogeneity among the results. It is worth noting that the regression model for this moderator did not explain any of the variability among the result tests.

Further evaluation of the administration routes and pregnancy state among the pooled CCRs were realized via sub-group analysis and meta-regression. As shown in Table 2, no significant difference was obtained in CCRs between pregnant and non-pregnant women. However, only 8 RCTs evaluated BV treatments among pregnant women. In addition, RCTs among non-pregnant women showed a low *p*-value via Egger’s test (*p* = 0.046) demonstrating heterogeneity among the results. Therefore, meta-regression was realized between CCRs of pregnant and non-pregnant women. Meta-regression models revealed no significant association between pregnancy and CCR (beta (β) = 0.2250, *SE* = 0.3384, *p* = 0.5060), neither between administration routes of different types of treatment nor CCR (*p* = 0.5248). However, in the pregnancy sub-group (k = 8), the CCR was higher with the oral administration when compared to the local application (88.2% *versus* 64.4%, respectively), but this was not statistically significant (*p* = 0.0797).

#### Network Analysis

The studies selected for the network meta-analysis showed comparisons between placebo and treatments or between treatments (see Table 3). The antibiotic treatments (AB) included 5-nitroimidazoles derivatives or clindamycin while the probiotic treatments (PB) included different lactobacilli, such as *Lactobacillus reuteri*, *L. gasseri*, *L. acidophilus*, *L. rhamnosus*, *L. brevis*, *L. salivarius*, *L. plantarum*, *L. fermentum*, and combinations between them. We classified the different therapies according to treatment type (AB or PB) and administration route (oral and local) by itself or combined therapies to avoid the generation of sub-networks.

As shown in Table 3, different treatments have been compared to placebo in many trials, appointing two therapies (“Oral AB (clindamycin) and Local AB (5-nitroimidazole) + PB” and “Oral AB (5-nitroimidazole) + PB”) as more far from control (“placebo”). It is important to mention that there were no multi-arm trials (trials with more than two arms) in our network, thus avoiding inference and incorrect correlations (data not shown). Further evaluation was realized through P-scores, allowing to generate a ranking of treatments from most to least beneficial among patients accordingly to CCRs. These P-scores measure the certainty that one treatment is better than other treatment averaged over all competing treatments. As shown in Table 3, the highest P-score was also achieved by the combined therapy of antibiotic by both administration routes plus local probiotic (oral AB (clindamycin) and local AB (5-nitroimidazole) + PB, P-score = 0.9208), followed by oral administration of antibiotic and probiotic (Oral AB(5-nitroimidazole) + PB, P-score = 0.8213), and local administration of antibiotic with oral probiotic (local AB (5-nitroimidazole) and oral PB, P-score = 0.6783). The first and second combined therapies demonstrated statistical significances (*p*-values of 0.0025 and 0.0096, respectively), when compared to the remaining BV treatments.

These results appointed to better effectiveness from orally combined therapies and local or oral administration of probiotics. However, when comparing the effectiveness outcomes between different treatments and placebo in trials, it was possible to observe treatments with considerable overlapping confidence intervals.

A clear-cut decision of the best BV treatment was not possible due to the heterogeneity of outcomes reported in the trials, indicating the necessity for more randomized controlled trials and a better characterization of the type of antibiotics and probiotics applied in BV treatment. 

### 2.5. Evaluation of Probiotic Therapy in BV Treatment

In the data set, the probiotic treatments contained a greater variability of different lactobacilli when compared to antibiotic treatments (5-nitroimidazoles derivatives or clindamycin). These probiotic lactobacilli were evaluated by themselves or combined with other probiotic species or antibiotics. As shown in Table 4, the number of lactobacilli species or strains showed statistically significant differences in the CCRs of BV treatment (*p* < 0.0001). Probiotic or combined therapies containing one or two lactobacilli demonstrated similar high CCRs and no statistically significant difference between them (*p* = 0.4455). However, CCR in BV treatment dropped in studies using three probiotic lactobacilli.

Several combinations of two and three lactobacilli species were evaluated among trials, *L. rhamnosus*, *L. reuteri*, *L. acidophilus*, and *L. gasseri* being the most frequently used species. However, no statistically significant differences were found among a specific combination of two and three lactobacilli. Furthermore, when analyzing *Lactobacillus* species individually, the absence of *L. gasseri* in the probiotic administration and the co-use of antibiotics with *L. acidophilus* showed higher CCRs in BV treatment demonstrating statistically significant differences (more exactly, *p* = 0.0051 and *p* < 0.0001, respectively). Finally, no correlation was found among CCRs of the remaining lactobacilli species.

### 2.6. The Geographical Disparity in CCR among BV Treatments 

The clinical cure rates of BV treatment among studies of different countries and regions substantially varied, as previously shown in Table 1. Therefore, a sub-group analysis was realized between the CCRs and the regions and countries with a minimum of published studies (see Table 5); more exactly, at least three studies per country and region. In both scenarios, statistically significant variations were detected on CCRs among studies in different countries (*p* = 0.0012) and regions (*p* < 0.0001). When comparing different regions, the highest CCRs were obtained from trials in Asia (90.0%) and Europe (71.1%). Meanwhile, North America and Africa showed the lowest average values of CCR (67.8 and 67.6%, respectively). However, when further analyzing the effectiveness of BV treatments in different countries, it is possible to observe discrepancies among the CCRs of Nigeria (78.0%) and Egypt (58.3%), being both previously lumped in as part of the Africa region. Likewise, European countries reported different CCRs in BV treatment, such as Sweden (55.7%), Belgium (64.4%), and Bulgaria (77.1%). Further analysis through meta-regression models attributed variability values of 17.1% among regions (*p* < 0.001). All eligible studies of North America belong to the USA. 

Egger’s linear regression test showed no publication bias among studies of Europe and Asia. However, Egger’s test was not realized in regions and countries with less than 10 studies (k < 10) due to a lack of statistical power. 

## 3. Discussion

This meta-analysis included a final set of 25 eligible studies with a total of 57 randomized controlled trials (RCTs), comparing the effectiveness of different types of BV treatments including non-pregnant and pregnant women. All treatments evaluated the clinical cure rates (CCRs) after initial treatment and the cumulative incidence of BV reinfection. The CCRs differences were analyzed between treatments (antibiotics, probiotics, and conjugates) and routes of administration (oral and local), assessing therapies with higher effectiveness in BV treatment.

### 3.1. Effectiveness of BV Treatments among Women

Initially, the highest CCRs in our data set were achieved by Hantoushzadeh et al. (96.20%) and Raja et al. (93.86%) among pregnant and non-pregnant women, respectively. In Iran, Hantoushzadeh et al. applied two different treatments in each group set of 250 pregnant women involving one probiotic treatment with the consumption of a mixed-lactobacilli yogurt and another antibiotic treatment with the oral ingestion of clindamycin (300 mg) [36]. No statistical differences were found among these treatments, and both showed CCRs above 90% (probiotic treatment: 238/250, and antibiotic treatment: 243/250). Meanwhile, in India, Raja et al. applied an oral antibiotic treatment with 500 mg of metronidazole (52/57) and 500 mg of tinidazole (55/57) [20]. However, a further evaluation of the 57 RCTs in our data set was realized through network meta-analysis, allowing us to identify certain therapies with better effectiveness in BV treatment. The best CCRs based on P-scores were an oral clindamycin and local 5-nitroimidazole + PB (P-score = 0.9208) and oral 5-nitroimidazole) + PB (P-score = 0.8213). The first type of treatment combined an antibiotic orally administrated (600 mg clindamycin) with a local administration of an antibiotic (1000 mg metronidazole) and a vaginal cream with probiotic lactobacilli (*L. acidophilus* and *L. rhamnosus* (1.00 × 10^9^ CFU) [38]). The second type of treatment administrated an oral antibiotic (such as 2000 mg of tinidazole, 1000 mg of metronidazole, 200 mg of ofloxacin, and 500 mg of ornidazole) with an oral probiotic (*L. rhamnosus* GR-1 and *L. reuteri* RC-14 (1.00 × 10^9^ CFU) (Anukam et al., 2006; Martinez et al., 2009) or *B. coagulans* (Ratna Sudha et al., 2012)). Anukam and colleagues applied in patients a combined therapy with oral metronidazole (500 mg, twice daily from days 1 to 7) and oral *L. rhamnosus* GR-1 plus *L. reuteri* RC-14 (twice daily from days 1 to 30), while Martinez and colleagues administrated a single dose of tinidazole (2000 mg) supplemented with two capsules containing *L. rhamnosus* GR-1 and *L. reuteri* RC-14 every morning for 4 weeks. Finally, Ratna Sudha and colleagues assigned a dose of antibiotic therapy (Ofloxacin–Ornidazole with 200–500 mg per capsule/day for 5 days along with vaginal co-kimaxazol peccaries for 3 days) simultaneously with two probiotic capsules (1.00 × 10^9^ CFU of *Bacillus coagulans* Unique IS-2 per capsule). 

On the other hand, the lowest average of CCR among pregnant women in our data set was reported by Darwish et al. (58.33%), which included four different treatments for pregnant women [21], those being: (1) an oral antibiotic treatment with 250 mg of metronidazole (27/39, 69.20%); (2) an oral antibiotic treatment with 300 mg of clindamycin (26/39; 66.70%); (3) a local antibiotic treatment with 500 mg of metronidazole (19/39, 48.70%); and (4) a local antibiotic treatment with 100 mg of clindamycin (19/39, 48.70%). Meanwhile, in the non-pregnant group, the lowest CCR was reported by Larsson et al. (46.85%), which included a combined treatment with oral clindamycin and local metronidazole plus the interaction of different strains of lactobacilli such as *L. rhamnosus*, *L. jensenii*, *L. gasseri*, and *L. crispatus.* [35]. In the same way, low CCRs in the BV treatment of non-pregnant women were also detected in non-combined therapies through a network meta-analysis. Based on P-scores, some low CCRs were found in certain treatments of local administration of antibiotics (*Local 5-nitroimidazole*, P-score = 0.2891; and *Local clindamycin*, P-score = 0.2681) when compared to placebo. 

Although general results in network meta-analysis indicated the local administration route as the preferential therapy for probiotic treatment, the average CCR was higher with oral administration when compared to local application among pregnant women despite the fact that no statistically significant differences were found. When analyzing the RCTs among pregnant women, the difference in both CCRs could be attributed to an oral probiotic treatment used by Hantoushzadeh et al. [36]. This study reported the best CCR among the sub-group set of trials on pregnant women, where Hantoushzadeh and colleagues administrated a probiotic yogurt (100 g twice a day for one week) containing *Lactobacillus bulgaricus*, *Lactobacillus acidophilus*, other probiotic lactobacilli, *Streptococcus thermophilus,* and *Bifidobacterium lactis* [36]. This probiotic yogurt was chosen due to the persistence of its probiotic bacteria in the gastrointestinal tract (resistance against bile and gastric acid), and its similarity to the common yogurts consumed in daily life. The probiotic yogurt contained 1.00 × 10^7^ CFU of *Lactobacillus acidophilus* per milliliter and the count was previously measured in De Man-Rogosa-Sharpe (MRS) Agar. 

### 3.2. Characterization of the Lactobacilli Species in Probiotic Therapies

Despite the diversity among probiotic treatments, most therapies use *Lactobacillus* species in the treatment of bacterial vaginosis through oral and local administration routes. As was previously referred to, our data set showed that these probiotic lactobacilli can be applied by themselves or combined with antibiotics (such as 5- nitroimidazole derivatives and clindamycin) or other probiotic species (such as *Bacillus coagulans*, *Streptococcus thermophilus*, and *Bifidobacterium lactis*) [21,35,36,43]. Several lactobacilli species were evaluated among the 57 RCTs of this meta-analysis, such as *L. acidophilus*, *L crispatus*, *L. rhamnosus*, *L. reuteri*, *L. delbrueckii*, *L. gasseri*, *L. fermentum*, *L. brevis*, *L. salivarius*, and *L. plantarum.* Although our sub-group analysis reported a non-statistical difference between treatments, the highest CCR was shown by probiotic treatment. Therefore, network meta-analysis was realized to identify treatments with higher effectiveness in BV treatment when compared to placebo assays (control). According to P-scores, treatments with the local or oral probiotic application have a higher P-score when compared to exclusively oral and local application of antibiotics. Based on this information it is important to characterize the lactobacilli species in probiotic and combined treatments. Although the number of lactobacilli on probiotic treatments showed statistically significant differences in the CCRs (*p* < 0.0001), this evaluation only considered *Lactobacillus* species. Probiotic products with one or two lactobacilli demonstrated higher CCRs in BV treatment. Additionally, combined therapies between antibiotics and two lactobacilli demonstrated high CCRs in BV treatment, such as *L. acidophilus* plus *L. rhamnosus* (79.7%) and *L. rhamnosus* plus *L. reuteri* (85.1%). In 2013, Kovachev and Dobrevski–Vacheva successively treated BV women with 600 mg of oral clindamycin, 1000 mg of local metronidazole, and the local application of *L. acidophilus* plus *L. rhamnosus* (1.00 × 10^9^ CFU), achieving a CCR of 87.5% [38]. Meanwhile, the effectiveness of the probiotic treatment with *L. rhamnosus* plus *L. reuteri* was evaluated by itself [39] and combined with antibiotic treatment (tinidazole and metronidazole) through local and oral administration routes [19,25,41]. Once again, the CCRs of the combined therapies (87.5–90.0%) surpassed the CCRs of the monotherapies with these lactobacilli combinations (61.5%), showing better outcomes when the probiotic treatment was applied through the local administration route. It is also important to mention that *L. gasseri* was present in five of the seven trials in combination with metronidazole and clindamycin as an aggressive treatment against BV [35]. However, Larsson and colleagues reported low CCRs in BV treatment (55.6%). Likewise, the probiotic combination of *L. rhamnosus* and *L. gasseri* showed the lowest CCR (63.0%) in our data set among combinations with two lactobacilli (Table 4). Finally, statistically significant differences were found (*p* = 0.0051) between the presence and the absence of *L. gasseri* in RCTs for BV treatment, showing a greater CCR among RCTs without this species. Another significant *p*-value was reported between the presence and the absence of *L. acidophilus* among combined therapies with antibiotics (*p* < 0.0001), evidencing higher CCRs in treatments with *L. acidophilus* (90.4%). However, further studies should evaluate the effectiveness of treatments with *L. acidophilus* plus antibiotics among BV women. Finally, several studies reported beneficial effects from combinations between lactobacilli and other bacteria in the probiotic activity against BV [17,36,43,45]. However, the combination of lactobacilli and other probiotic bacteria was not evaluated in the present meta-analysis due to the lack of information in RCTs. Several authors already stated the need to fully characterize probiotic species and to take into account probiotic formulation [46,47]. In 2020, Pat et al. emphasized the necessity to further characterize the common and unique functional properties of vaginal and intestinal probiotics, the findings of which should guide to the rational formulation of next-generation probiotics for intestinal and vaginal health. Therefore, further studies should evaluate possible synergetic interactions of multiple microbial species on RCTs in BV treatments.

### 3.3. The Geographical Disparity in CCR among BV Treatments 

The clinical cure rates of BV treatment among countries markedly varied due to different therapies. As previously shown in Table 5, the average CCRs among regions and countries demonstrated statistically significant differences (*p* = 0.0085 and *p* = 0.0069, respectively). These discrepancies on CCRs were easily detected among several countries, such as Sweden, Egypt, India, and Nigeria. Sweden and Egypt evidenced the lowest average of CCRs in RCTs, while India and Nigeria showed the highest average of CCRs.

In Sweden, these trials with low CCRs evaluated combined treatments through oral clindamycin, the gel application of metronidazole, and vaginal gelatin capsules containing different mixtures of lactobacilli at 1.00 × 10^9^ CFU [35]. The lactobacilli mixtures were the following: (1) *L. gasseri* plus *L. rhamnosus*; (2) two strains of *L. crispatus* plus *L. gasseri*; (3) and two strains of *L. gasseri* plus *L. rhamnosus*. In addition, two more trials were conducted through oral capsules containing two strains of *L. gasseri* plus *L. rhamnosus* and *L. rhamnosus* plus *L. reuteri* [35]. In these trials, combined treatments using *L. gasseri* showed the lowest CCRs and, therefore, our meta-analysis evidenced statistically significant differences in the effectiveness of BV treatment when using this *Lactobacillus* sp. However, further analysis of *L. gasseri* in other RTCs should be realized in future studies to clarify these data. In Egypt, Darwish and colleagues realized monotherapies among BV women leading to low CCRs [21]. These authors evaluated four types of BV treatment. More exactly, two treatments consisted of the oral administration of capsules containing 250 mg metronidazole or 300 mg clindamycin. The remaining two treatments applied a local administration of vaginal suppositories with 500 mg of metronidazole or a vaginal cream with 100 mg of clindamycin [21]. No combined therapies were applied in BV treatment and antibiotic treatments used low concentrations of antibiotic when compared to others RCTs with higher CCRs, indicating possibly an inappropriate application and non-optimal concentration of both antibiotics. 

On the other hand, RCTs in India reported high CCRs applying higher doses of antibiotics orally applied through capsules of metronidazole (500 and 2000 mg), tinidazole (500 and 2000 mg), ornidazole (1500 mg), and secnidazole (2000 mg) [20,29]. However, in Nigeria, Anukam and colleagues studied antibiotic, probiotic, and combined treatments among BV women. Antibiotic treatments evidenced the lowest CCRs in their population set, showing 60% of CCR with a vaginal gel of metronidazole (37.5 mg) and 76% of CCR with an oral administration of metronidazole (1000 mg). Next, probiotic treatment included the local application of *L. rhamnosus* and *L. reuteri* at 1.00 × 10^9^ CFU through a vaginal suppository. Finally, the combined treatment showed the highest values of CCR by orally applying metronidazole (1000 mg) and *L. rhamnosus* plus *L. reuteri* (1.00 × 10^9^ CFU) [19,41]. These trials suggested that combined therapies between antibiotic and probiotic treatments could lead to high CCRs in BV and the reduction of the optimal concentration of the applied antibiotics.

## 4. Materials and Methods

### 4.1. Data Selection, Search Strategy, and Study Guidelines

This study was conducted following preferred reporting items for systematic reviews and meta-analyses (PRISMA) strategies [48]. Scopus, PubMed, and Cochrane Library databases were searched for English papers using the following medical subject heading terms (MESH): “bacterial vaginosis”; “treatment”; “probiotic”; “antibiotic”; and “cure rate”. No restrictions on the year of study or the participants’ ages were imposed.

In each electronic database, a combination of MESH terms was used to conduct the search applying the following strategy (for example, in the MEDLINE): ‘‘(“Bacterial Vaginosis”) AND (Treatment) AND (“Cure rate”)’’. All studies published until 30 December 2020 were retrieved. The articles reporting the clinical cure rate, type of treatment, administration route, and place of study were included. The references of all included studies were also checked in order to find additional records. The search was limited to human clinical control trials. All references were compiled into a database Mendeley Library and then managed using Excel.

### 4.2. Screening Process

Duplicates were initially identified and eliminated in Mendeley after entering all the recognized studies into an Excel self-created database (see General screening.xlsx in supplementary file). All articles were assessed by one reviewer (AMM-B) by screening titles, abstracts, topics, and, finally, full texts. An additional examination of the selected articles was realized by a second author (AM) focused on the homogeneity of the eligibility criteria of both reviewers in the initial data set. Discrepancies were resolved by discussion between all authors before finalizing the records for the evaluation of eligibility criteria.

### 4.3. Eligibility Criteria

Reviews, editorials, congress or meeting abstracts, literature in languages other than English, case reports, clinical trials, and letters to editors were excluded from the final data set. Duplicate reports on different databases and studies with unclear and missing data were also omitted. 

### 4.4. Data Extraction and Quality Assessment

Methodological quality assessment of the studies was performed using a checklist for necessary items as outlined in the critical appraisal skills program (CASP) checklists [49]. For each article, a series of critical questions were asked. If the pertinent data were given, the question was scored as ‘‘yes”. If there was any doubt or no information in the study, that question was marked as ‘‘no’’. A data extraction form was designed to extract the relevant characteristics of each study. The extracted information included the authors’ names, time of the study, year of publication, location, sample size, clinical curation rate, and type of treatment (such as antibiotics, probiotics, and conjugates). The first author (AMM-B) extracted all data, further confirmation and final evaluation were realized by the remaining authors (AM, ET, and FSC-M).

### 4.5. Data Analysis and Statistical Methods

Meta-analysis was performed using the RStudio software (Version 1.4.1103; https://rstudio.com/; accessed on 4 February 2021), using several R packages (meta, metafor, dmetar, poibin, stringr, and netmeta). The clinical cure rates were computed, and values were reported with confidence intervals (CI) of 95%. The heterogeneity was assessed by the Cochrane Q and I^2^ tests. Considering the heterogeneity indices, the random-effects model was used and the logit transformation was applied to calculate the pooled frequencies. Sub-group analysis and meta-regression were performed according to the type of treatment, pregnancy status, and geographic distribution. Outliers’ analysis was done with the Baujat diagram. Egger test, funnel plot, and *p*-curve analyses were used to explore publication bias. As recommended by Sterne and colleagues [50], funnel plot asymmetry tests were only performed when the number of studies was at least ten (k ≥ 10). All *p*-values < 0.05 were considered statistically significant, except for Egger’s test (*p* < 0.10) [51]. A network meta-analysis was used to compare the efficacy of all pairs of interventions that included placebo, antibiotic, probiotic, and conjugate or combined treatments. The random-effects model was used in sub-group analyses. Odds ratios (OR) were used to report the effect size for assessing efficacy. In addition, inconsistency between direct and indirect evidence was evaluated based on the Z test and provided a *p*-value to indicate inconsistency (*p* < 0.05). Treatment efficacy rank was determined by P-scores in a manner that the larger P-score suggested a better treatment based on efficacy.

## 5. Conclusions

In summary, this meta-analysis allowed for the characterization of patterns of CCRs in BV treatment and, consequently, the identification of better therapies. Certain combined therapies could surpass monotherapies in the effectiveness of BV treatment among women, appointing for a combination of antibiotics and probiotic lactobacilli through oral and/or local administration routes. It is important to mention that not all combined therapies between antibiotics and probiotics are efficient treatments among BV women. Several variables (such as lactobacilli species and concentration, administration route, time and phases of treatment, and the optimal concentration of antibiotics) should be considered in the formulation of BV treatments. The combined therapies also appointed to the reduction of the optimal concentration of antibiotics. Double phase treatments of antibiotics on women suggested an increment of CCR in BV women. Although the present meta-analysis was performed methodically, there are some limitations in this study: (1) heterogeneity exists in some sub-group and overall analyses; (2) characterization based on host epidemiological factors (age, ethnic groups, and other characteristics) with different BV treatments could not be assessed; and (3) a detailed analysis of more specific subcategories (such as different probiotic bacteria) was not possible. These limitations are due to a lack of sufficient published data. Published data other than in English and in vitro or in vivo assays were not incorporated in this meta-analysis. Additionally, the authors did not contact any corresponding author of the studies with missing data for further clarity, and so several reports were discharged from the final data set. Future studies should assess the formulation of new combined therapies to improve BV treatment.

## Figures and Tables

**Table 1:** General information extracted from the data set and selected for the present meta-analysis.

First Author, Year	Region	Country	Age Group (Years)	Pregnancy	Clinical Cure Rate (%)	Treatment Assays *	References
Raja, 2016	Asia	India	18–51	No	107/114 (93.86)	AB	[20]
Darwish, 2007	Africa	Egypt	20–27	Yes	91/156 (58.33)	AB	[21]
Ling, 2012	Asia	China	NR	No	45/55 (81.81)	AB, PB	[22]
Larsson, 2008	Europe	Norway	18–53	No	24/37 (64.86)	AB + PB	[23]
Kekki, 2002	Europe	Finland	17–43	Yes	123/187 (65.77)	AB	[24]
Martínez, 2009	America	Brazil	16–51	No	44/64 (68.75)	AB, AB + PB	[25]
Voorspoels, 2002	Europe	Belgium	NR	No	49/76 (64.47)	AB	[26]
Brandt, 2008	Europe	Germany	18–50	No	240/263(91.25)	AB	[27]
Schwebke, 2011	America	USA	21–35	No	168/287 (58.53)	AB	[28]
Thulkar, 2012	Asia	India	20–40	No	304/344 (88.37)	AB	[29]
Eriksson, 2005	Europe	Sweden, Finland & Norway	18–53	No	111/187 (59.35)	AB, AB + PB	[30]
Schwebke, 2015	America	USA	20–35	No	144/308 (46.75)	AB	[31]
Paavonen, 2000	Europe	Finland	16–60	No	172/233(73.82)	AB	[32]
Kurkinen, 2000	Europe	Finland	22–34	Yes	54/62 (87.09)	AB	[33]
Sobel, 2001	America	USA	16–58	No	270/342 (78.94)	AB	[34]
Larsson, 2011	Europe	Sweden	19–55	No	35/63 (55.55)	AB + PB	[35]
Hantoushzadeh, 2012	Asia	Iran	23–33	Yes	481/500 (96.20)	AB, PB	[36]
Kovachev, 2013a	Europe	Bulgaria	NR	No	485/539 (89.98)	AB, AB + PB	[37]
Kovachev, 2013b	Europe	Bulgaria	NR	No	224/381 (58.79)	AB, PB, AB + PB	[38]
Vujic, 2013	Europe	Croatia	18–58	No	243/395 (61.52)	PB	[39]
Anukam, 2006a	Africa	Nigeria	18–50	No	30/40 (75.00)	AB, PB	[19]
Bradshaw, 2012	Oceania	Australia	18–50	No	381/408 (93.38)	AB, AB + PB	[40]
Anukam, 2006b	Africa	Nigeria	18–44	No	82/106 (77.35)	AB, AB + PB	[41]
Mastromarino, 2009	Europe	Italy	23–45	No	12/18 (66.66)	PB	[18]
Marcone, 2008	Europe	Italy	18–40	No	63/84 (75.00)	AB, AB + PB	[42]
Ratna, 2011	Asia	India	30–36	No	25/40 (62.50)	AB, AB + PB	[43]
Bohbot, 2018	Europe	France	NR	No	52/76 (68.42)	AB, AB + PB	[44]

* AB: Antibiotic, PB: probiotic, AB + PB: Conjugate or combined therapies. NR: Not reported. The clinical cure rate was calculated with 95% CI through random-model and significance level ≤0.05 (*p*-value). The sample size and prevalence were used to calculate the combined clinical cure rate. The complementary proportion of each study was considered as reinfection or non-cure.

**Table 2:** Pooled CCR of treatments for bacterial vaginosis among pregnant and non-pregnant women.

Treatment	k = 57 (Trials)	Clinical Cure Rate (95% CI)	Egger’s Test	Random Effects Model			
			*p* *	t	Q	I^2^	*p* **
Only antibiotics	35	74.6 (69.1–79.3)	0.091	0.7396	283.42	88.0	0.8453
Conjugate (antibiotic + probiotic)	16	74.1 (63.1–82.7)	0. 296	0.9101	89.10	83.2	
Only probiotics	6	79.7 (59.3–91.4)	-	1.1347	105.03	95.2	
**Pregnancy**							
No	49	74.3 (69.4–78.6)	0.046	0.7554	385.57	87.6	0.5946
Yes	8	78.6 (61.0–89.6)	-	1.1908	122.26	94.3	

The trials considered (k = 57) from 25 studies. * Egger’s test was not realized for treatments with less than 10 trials (k < 10) due to lack of statistical power in the detection of publication bias. ** Test for sub-group difference.

**Table 3:** P-scores ranked from different types of treatments for bacterial vaginosis.

Treatment	P-Score	Odds Ratio (95 % IC)	*p* *
Oral AB (clindamycin) and Local AB (5-nitroimidazole) + PB	0.9208	44.4355 (3.8078; 518.5520)	0.0025
Oral AB (5-nitroimidazole) + PB	0.8213	19.0430 (2.0464; 177.2109)	0.0096
Local AB (5-nitroimidazole) and Oral PB	0.6783	9.7905 (0.6850; 139.9286)	0.0927
Oral AB (clindamycin) and Local AB (5-nitroimidazole)	0.5757	6.7642 (0.2703; 169.2481)	0.2446
Oral AB (5-nitroimidazole) and Local PB	0.5561	5.4659 (0.3630; 82.2974)	0.2196
Only local PB	0.5431	4.7222 (1.2726; 17.5231)	0.0203
Only oral PB	0.5102	4.3564 (0.5799; 32.7269)	0.1526
Local AB (clindamycin) + PB	0.4856	3.9458 (0.3049; 51.0635)	0.2934
Only oral AB (clindamycin)	0.4802	3.8568 (0.2989; 49.7715)	0.3009
Local AB (5-nitroimidazole) + PB	0.4551	3.5051 (0.3416; 35.9620)	0.2910
Only oral AB (5-nitroimidazole)	0.3188	2.1864 (0.4660; 10.2584)	0.3213
Only local AB (5-nitroimidazole)	0.2891	2.0026 (0.6686; 5.9983)	0.2147
Only local AB (clindamycin)	0.2681	1.8320 (0.4772; 7.0333)	0.3778
Placebo	-	-	-

AB: Antibiotic, PB: probiotic, AB + PB: Conjugate or combined therapies. * Test for sub-group difference.

**Table 4:** Sub-group analysis of the efficacy in BV treatment with probiotic lactobacilli.

Number of Lactobacilli Species ^a^	k	CCR (95% CI)	Random Effects Model			
			T	Q	I^2^	*p* *
1 ^b^	4	82.6 (74.5–88.5)	0	2.89	0	<0.0001
2	10	77.3 (62.9–87.3)	1.0376	145.15	93.8	
3	7	56.5 (48.5–64.2)	0	3.72	0.0	
Combinations (2 strains)						
*L. rhamnosus* + *L. acidophilus*	3	79.7 (37.4–96.3)	1.6510	115.94	98.3	0.2413
*L. rhamnosus* + *L. gasseri*	2	63.0 (48.3–75.6)	0	0.27	0.0	
*L. rhamnosus* + *L. reuteri*	5	80.8 (62.0–91.6)	0.9469	23.62	83.1	
Combinations (3 strains)						
*L. crispatus* + *L. gasseri* + *L. jensenii*	2	45.9 (22.5–71.4)	0.4557	1.52	34.4	0.6728
*L. rhamnosus* + *L. gasseri* (2 strains)	2	61.3 (33.9–83.1)	0	0.32	0.0	
Other ^c^	3	57.9 (48.9–66.5)	0	0.71	0.0	
Includes *L. rhamnosus*?						
No	7	70.0 (53.9–82.4)	0.7112	15.14	60.4	0.6010
Yes	14	74.8 (63.1–83.7)	0.9333	155.50	91.6	
*L. rhamnosus* with antibiotics?						
Yes	11	77.4 (64.4–86.7)	0.9548	74.43	86.6	0.1323
No	3	61.3 (42.0–77.5)	0.6031	21.55	90.7	
Includes *L. reuteri*?						
No	15	71.8 (57.1–83.0)	1.1589	136.89	89.8	0.6953
Yes	6	75.3 (62.2–84.9)	0.6225	26.31	81.0	
*L. reuteri* with antibiotics?						
Yes	4	77.6 (52.9–91.4)	1.0339	19.86	84.9	0.9496
No	2	76.4 (38.0–94.5)	1.1000	5.27	81.0	
Includes *L. acidophilus*?						
No	18	71.4 (63.8–78.0)	0.5355	49.68	65.8	0.6427
Yes	3	79.7 (37.4–96.3)	1.6510	115.94	98.3	
*L. acidophilus* with antibiotics?						
Yes	2	90.4 (84.9–94.1)	0.2775	2.20	54.5	<0.0001
No	1	42.7 (34.8–50.9)	-	0.00	-	
Includes *L. gasseri*?						
No	14	79.3 (68.7–87.0)	0.9565	173.70	92.1	0.0051
Yes ^d^	7	58.4 (47.8–68.2)	0	3.89	0.0	

^a^ One study was discarded because it did not provide information about probiotic species [16]. ^b^ *L crispatus*, *L. rhamnosus*, *L. delbrueckii*, and *B. coagulans* (k = 1). ^c^ Other combinations include: *L. crispatus* (two strains), and *L. gasseri*; *L. rhamnosus*, *L. gasseri*, and *L. fermentum*; *L. brevis*, *L. salivarius*, and *L. plantarum* (k = 1). ^d^ Every treatment with *L. gasseri* was conducted with antibiotics. * Test for sub-group difference.

**Table 5:** Sub-group analysis of the efficacy in BV treatment in different regions and countries.

Sub-Groups Region (≥3 Studies) ^a^	k (Trials)	Clinical Cure Rate (95% CI) (%)	Egger’s Test	Random Model			
			*p* *	t	Q	I^2^	*p* **
Europe	30	71.1 (64.4–76.9)	0.378	0.7551	267.62	89.2	0.0085
Asia	12	90.0 (81.7–94.8)	0.298	1.1241	90.56	87.9	
Africa	8	67.6 (56.1–77.4)	-	0.5882	24.73	71.7	
North America	5	67.8 (56.4- 77.4)	-	0.5170	30.87	87.0	
**Country (≥3 studies) ^a^**							
India	8	87.9 (76.8–94.1)	-	1.0057	44.37	84.2	0.0069
Sweden	7	55.7 (43.0–67.7)	-	0.0	3.33	0.0	
Bulgaria	5	77.1 (51.9–91.3)	-	1.2791	166.01	97.6	
Egypt	4	58.3 (47.0–68.8)	-	0.3257	5.90	49.1	
Nigeria	4	78.0 (59.4–89.5)	-	0.7580	11.65	74.2	
Italy	3	72.9 (59.6–83.1)	-	0.3423	3.41	41.3	
Belgium	3	64.4 (53.1–74.3)	-	0.0	0.24	0.0	
USA	5	67.8 (56.4–77.4)	-	0.5170	30.87	87.0	

^a^ Multi-region & multi-country studies were discarded in this analysis [17,18]. * Egger’s test was not realized in regions and countries with less than 10 studies (k < 10) due to a lack of statistical power in the detection of publication bias. ** Test for sub-group differences.

## Data Availability

All data presented in this study are available on request by contacting the corresponding author.

## Supplementary Materials

The following are available online at https://www.mdpi.com/article/10.3390/antibiotics10080978/s1, Excel data set S1: General screening.xlsx.

## References

[1] Romero D., Andreu A. (2016). Vaginosis bacteriana. Enferm. Infecc. Microbiol. Clin..

[2] Sobel J. (2000). Bacterial vaginosis. Annu. Rev. Med..

[3] Tamrakar R., Yamada T., Furuta I., Cho K., Morikawa M., Yamada H., Sakuragi N., Minakami H. (2007). Association between Lactobacillus species and bacterial vaginosis-related bacteria, and bacterial vaginosis scores in pregnant Japanese women. BMC Infect. Dis..

[4] Jones A. (2019). Bacterial Vaginosis: A Review of Treatment, Recurrence, and Disparities. J. Nurse Pract..

[5] Kumar N., Behera B., Sagiri S.S., Pal K., Ray S.S., Roy S. (2011). Bacterial vaginosis: Etiology and modalities of treatment-A brief note. J. Pharm. Bioallied Sci..

[6] Woodman Z. (2016). Can one size fit all? Approach to bacterial vaginosis in sub-Saharan Africa. Ann. Clin. Microbiol. Antimicrob..

[7] Petrova M.I., Lievens E., Malik S., Imholz N., Lebeer S. (2015). Lactobacillus species as biomarkers and agents that can promote various aspects of vaginal health. Front. Physiol..

[8] Borgdorff H., van der Veer C., van Houdt R., Alberts C.J., de Vries H.J., Bruisten S.M., Snijder M.B., Prins M., Geerlings S.E., van der Loeff M.F.S. (2017). The association between ethnicity and vaginal microbiota composition in Amsterdam, The Netherlands. PLoS ONE.

[9] Chen H.M., Chang T.H., Lin F.M., Liang C., Chiu C.M., Yang T.L., Yang T., Huang C.Y., Cheng Y.N., Chang Y.A. (2018). Vaginal microbiome variances in sample groups categorized by clinical criteria of bacterial vaginosis. BMC Genom..

[10] Foessleitner P., Kiss H., Deinsberger J., Ott J., Zierhut L., Rosta K., Falcone V., Farr A. (2021). Screening Pregnant Women for Bacterial Vaginosis Using a Point-of-Care Test: A Prospective Validation Study. J. Clin. Med..

[11] Joseph R.J., Ser H.-L., Kuai Y.-H., Tan L.T.-H., Arasoo V.J.T., Letchumanan V., Wang L., Pusparajah P., Goh B.-H., Ab Mutalib N.-S. (2021). Finding a Balance in the Vaginal Microbiome: How Do We Treat and Prevent the Occurrence of Bacterial Vaginosis?. Antibiotics.

[12] Hainer B.L., Gibson M.V. (2011). Vaginitis: Diagnosis and treatment. Am. Fam. Physician.

[13] Amsel R., Totten P.A., Spiegel C.A., Chen K.C.S., Eschenbach D., Holmes K.K. (1983). Nonspecific vaginitis. Diagnostic criteria and microbial and epidemiologic associations. Am. J. Med..

[14] Donders G.G., Zodzika J., Rezeberga D. (2014). Treatment of bacterial vaginosis: What we have and what we miss. Expert Opin. Pharmacother..

[15] Van de Wijgert J.H.H.M., Borgdorff H., Verhelst R., Crucitti T., Francis S., Verstraelen H., Jespers V. (2014). The vaginal microbiota: What have we learned after a decade of molecular characterization?. PLoS ONE.

[16] Beigi R.H., Austin M.N., Meyn L.A., Krohn M.A., Hillier S.L. (2004). Antimicrobial resistance associated with the treatment of bacterial vaginosis. Am. J. Obstet. Gynecol..

[17] Mastromarino P., Vitali B., Mosca L. (2013). Bacterial vaginosis: A review on clinical trials with probiotics. New Microbiol..

[18] Mastromarino P., Macchia S., Meggiorini L., Trinchieri V., Mosca L., Perluigi M., Midulla C. (2009). Effectiveness of Lactobacillus-containing vaginal tablets in the treatment of symptomatic bacterial vaginosis. Clin. Microbiol. Infect..

[19] Anukam K.C., Osazuwa E., Osemene G.I., Ehigiagbe F., Bruce A.W., Reid G. (2006). Clinical study comparing probiotic Lactobacillus GR-1 and RC-14 with metronidazole vaginal gel to treat symptomatic bacterial vaginosis. Microbes Infect..

[20] Raja I.M., Basavareddy A., Mukherjee D., Meher B.R. (2016). Randomized, double-blind, comparative study of oral metronidazole and tinidazole in treatment of bacterial vaginosis. Indian J. Pharmacol..

[21] Darwish A., Elnshar E.M., Hamadeh S.M., Makarem M.H. (2007). Treatment options for bacterial vaginosis in patients at high risk of preterm labor and premature rupture of membranes. J. Obstet. Gynaecol. Res..

[22] Ling Z., Liu X., Chen W., Luo Y., Yuan L., Xia Y., Nelson K.E., Huang S., Zhang S., Wang Y. (2013). The Restoration of the Vaginal Microbiota After Treatment for Bacterial Vaginosis with Metronidazole or Probiotics. Microb. Ecol..

[23] Larsson P.G., Stray-Pedersen B., Ryttig K.R., Larsen S. (2008). Human lactobacilli as supplementation of clindamycin to patients with bacterial vaginosis reduce the recurrence rate; a 6-month, double-blind, randomized, placebo-controlled study. BMC Womens Health.

[24] Kekki M. (2001). Vaginal clindamycin in preventing preterm birth and peripartal infections in asymptomatic women with bacterial vaginosis: A randomized, controlled trial. Obstet. Gynecol..

[25] Martinez R.C.R., Franceschini S.A., Patta M.C., Quintana S.M., Gomes B.C., de Martinis E.C.P., Reid G. (2009). Improved cure of bacterial vaginosis with single dose of tinidazole (2 g), Lactobacillus rhamnosus GR-1, and Lactobacillus reuteri RC-14: A randomized, double-blind, placebo-controlled trial. Can. J. Microbiol..

[26] Voorspoels J., Casteels M., Remon J.P., Temmerman M. (2002). Local treatment of bacterial vaginosis with a bioadhesive metronidazole tablet. Eur. J. Obstet. Gynecol. Reprod. Biol..

[27] Brandt M., Abels C., May T., Lohmann K., Schmidts-Winkler I., Hoyme U.B. (2008). Intravaginally applied metronidazole is as effective as orally applied in the treatment of bacterial vaginosis, but exhibits significantly less side effects. Eur. J. Obstet. Gynecol. Reprod. Biol..

[28] Schwebke J.R., Desmond R.A. (2011). Tinidazole vs. metronidazole for the treatment of bacterial vaginosis. Am. J. Obstet. Gynecol..

[29] Thulkar J., Kriplani A., Agarwal N. (2012). A comparative study of oral single dose of metronidazole, tinidazole, secnidazole and ornidazole in bacterial vaginosis. Indian J. Pharmacol..

[30] Eriksson K., Carlsson B., Forsum U., Larsson P.G. (2005). A double-blind treatment study of bacterial vaginosis with normal vaginal lactobacilli after an open treatment with vaginal clindamycin ovules. Acta Derm. Venereol..

[31] Schwebke J.R., Desmond R.A. (2015). A randomized trial of the duration of therapy with metronidazole plus or minus azithromycin for treatment of symptomatic bacterial vaginosis. Clin. Infect. Dis..

[32] Paavonen J., Mangioni C., Martin M.A., Wajszczuk C.P. (2000). Vaginal clindamycin and oral metronidazole for bacterial vaginosis: A randomized trial. Obstet. Gynecol..

[33] Kurkinen-Räty M., Vuopala S., Koskela M., Kekki M., Kurki T., Paavonen J., Jouppila P. (2000). A randomised controlled trial of vaginal clindamycin for early pregnancy bacterial vaginosis. Br. J. Obstet. Gynaecol..

[34] Sobel J., Peipert J.F., McGregor J.A., Livengood C., Martin M., Robbins J., Wajszczuk C.P. (2001). Efficacy of clindamycin vaginal ovule (3-day treatment) vs. clindamycin vaginal cream (7-day treatment) in bacterial vaginosis. Infect. Dis. Obstet. Gynecol..

[35] Larsson P.G., Brandsborg E., Forsum U., Pendharkar S., Andersen K.K., Nasic S., Hammarström L., Marcotte H. (2011). Extended antimicrobial treatment of bacterial vaginosis combined with human lactobacilli to find the best treatment and minimize the risk of relapses. BMC Infect. Dis..

[36] Hantoushzadeh S., Golshahi F., Javadian P., Khazardoost S., Aram S., Hashemi S., Mirarmandehi B., Borna S. (2012). Comparative efficacy of probiotic yoghurt and clindamycin in treatment of bacterial vaginosis in pregnant women: A randomized clinical trial. J. Matern. Neonatal Med..

[37] Kovachev S., Vatcheva-Dobrevski R. (2013). Efficacy of combined 5-nitroimidazole and probiotic therapy of bacterial vaginosis: Randomized open trial. Akusherstvo I Ginekologiia.

[38] Kovachev S., Dobrevski-Vacheva R. (2013). Probiotic monotherapy of bacterial vaginosis: A open, randomized trial. Akusherstvo I Ginekol..

[39] Vujic G., Jajac Knez A., Despot Stefanovic V., Kuzmic Vrbanovic V. (2013). Efficacy of orally applied probiotic capsules for bacterial vaginosis and other vaginal infections: A double-blind, randomized, placebo-controlled study. Eur. J. Obstet. Gynecol. Reprod. Biol..

[40] Bradshaw C.S., Pirotta M., de Guingand D., Hocking J.S., Morton A.N., Garland S.M., Fehler G., Morrow A., Walker S., Vodstrcil L.A. (2012). Efficacy of oral metronidazole with vaginal clindamycin or vaginal probiotic for bacterial vaginosis: Randomised placebo-controlled double-blind trial. PLoS ONE.

[41] Anukam K., Osazuwa E., Ahonkhai I., Ngwu M., Osemene G., Bruce A.W., Reid G. (2006). Augmentation of antimicrobial metronidazole therapy of bacterial vaginosis with oral probiotic Lactobacillus rhamnosus GR-1 and Lactobacillus reuteri RC-14: Randomized, double-blind, placebo controlled trial. Microbes Infect..

[42] Marcone V., Calzolari E., Bertini M. (2008). Effectiveness of vaginal administration of Lactobacillus rhamnosus following conventional metronidazole therapy: How to lower the rate of bacterial vaginosis recurrences. New Microbiol..

[43] Ratna Sudha M., Yelikar K.A., Deshpande S. (2012). Clinical Study of Bacillus coagulans Unique IS-2 (ATCC PTA-11748) in the Treatment of Patients with Bacterial Vaginosis. Indian J. Microbiol..

[44] Bohbot J.M., Daraï E., Bretelle F., Brami G., Daniel C., Cardot J.M. (2018). Efficacy and safety of vaginally administered lyophilized Lactobacillus crispatus IP 174178 in the prevention of bacterial vaginosis recurrence. J. Gynecol. Obstet. Hum. Reprod..

[45] Ruiz F.O., Gerbaldo G., Asurmendi P., Pascual L.M., Giordano W., Barberis I.L. (2009). Antimicrobial Activity, Inhibition of Urogenital Pathogens, and Synergistic Interactions between Lactobacillus Strains. Curr. Microbiol..

[46] Altermann E., Klaenhammer T.R. (2011). Group-specific comparison of four lactobacilli isolated from human sources using differential blast analysis. Genes Nutr..

[47] Pan M., Hidalgo-Cantabrana C., Goh Y.J., Sanozky-Dawes R., Barrangou R. (2020). Comparative Analysis of Lactobacillus gasseri and Lactobacillus crispatus Isolated From Human Urogenital and Gastrointestinal Tracts. Front. Microbiol..

[48] Liberati A., Altman D.G., Tetzlaff J., Mulrow C., Gøtzsche P.C., Ioannidis J.P.A., Clarke M., Devereaux P.J., Kleijnen J., Moher D. (2009). The PRISMA statement for reporting systematic reviews and meta-analyses of studies that evaluate health care interventions: Explanation and elaboration. PLoS Med..

[49] Zeng X., Zhang Y., Kwong J.S.W., Zhang C., Li S., Sun F., Niu Y., Du L. (2015). The methodological quality assessment tools for preclinical and clinical studies, systematic review and meta-analysis, and clinical practice guideline: A systematic review. J. Evid. Based. Med..

[50] Sterne J.A.C., Sutton A.J., Ioannidis J.P.A., Terrin N., Jones D.R., Lau J., Carpenter J., Rücker G., Harbord R.M., Schmid C.H. (2011). Recommendations for examining and interpreting funnel plot asymmetry in meta-analyses of randomised controlled trials. BMJ.

[51] Lin L., Chu H. (2018). Quantifying publication bias in meta-analysis. Biometrics.

