# Supplementary material for: Comparative Effectiveness of Treatments for Bacterial Vaginosis: A Network Meta-Analysis
Source: Antibiotics (Basel). 2021 Aug 13;10(8):978. doi: 10.3390/antibiotics10080978 (PMC8388924; doi:10.3390/antibiotics10080978)
Supplement: Supplementary file 1 [file antibiotics-10-00978-s001.zip › antibiotics-1293936-supplementary.pdf]

Author	Country	Region	Ethnicity
Ratna_M_&_Yelikar_K_2011	India	Asia	Other
Kovachec_S_&Dobrevski_R_2013a	Bulgaria	Europe	Other
Larsson_P_ et_ al_2008	Norway	Europe	caucasian
Darwish_A_2007b	Egypt	Africa	Other
Brandt_et_ al_2008	Germany	Europe	caucasian
Darwish_A_2007a	Egypt	Africa	Other
Anukam_K_et_al_2006a	Nigeria	Africa	Black
Ling_Z_2012	China	Asia	Other
Kovachec_S_&Dobrevski_R_2013b1	Bulgaria	Europe	Other
Paavonen_J_et_al_2000	-	Europe	Other
Anukam_K_et_al_2006b	Nigeria	Africa	Black
Martínez_et_al_2009	Brazil	America	Other
Marcone_V_et_al_2008	Italy	Europe	Other
Kovachec_S_&Dobrevski_R_2013b2	Bulgaria	Europe	Other
Voorspoels_J_ et_ al_2002a	Belgium	Europe	caucasian
Voorspoels_J_ et_ al_2002b	Belgium	Europe	caucasian
Voorspoels_J_ et_ al_2002c	Belgium	Europe	caucasian
Kekki_M_ et_ al_2002	Finland	Europe	caucasian
Kurkinen_M_et_al_2000	Finlandia	Europe	caucasian
Eriksson_K_et_al_2005	Sweden_Finland_Nor	Europe	caucasian
Mastromarino_P_et_al_2009	Italy	Europe	Other
Vujic_G_et_al_2013	Croatia	Europe	Other

Pregnancy	events_tx1	n_tx1	events_tx2	n_tx2	TE
NO	9	20	16	20	-1.586965057
NO	274	297	211	242	0.559762961
NO	24	37	18	39	0.767255153
SI	27	39	19	39	0.862223511
NO	116	129	124	134	-0.329055639
SI	26	39	19	39	0.744440475
NO	8	18	15	17	-2.238046572
NO	21	30	24	25	-2.33075597
NO	98	112	61	143	2.241755532
NO	85	108	87	114	0.137085788
NO	43	49	23	57	2.360306955
NO	28	32	16	32	1.945910149
NO	28	42	35	42	-0.916290732
NO	98	112	65	126	1.882396743
NO	16	25	7	24	1.46266734
NO	16	26	7	24	1.357306824
NO	17	25	7	24	1.641074997
SI	123	187	64	188	1.314699754
SI	54	62	57	61	-0.747214402
NO	51	91	60	96	-0.267879445
NO	11	18	3	16	1.918322193
NO	243	395	40	149	1.471649351

sE	tx1_lab
0.717300636	Only local AB
0.290043612	Local AB+PB
0.47091862	Local AB+PB
0.472230822	Only oral AB
0.440009412	Only oral AB
0.466922043	Only oral AB
0.889756521	Only local AB
1.095626225	Only local AB
0.33199581	Oral AB and Local AB+PB
0.32214012	Only local AB
0.512652422	Oral AB+PB
0.640869944	Oral AB+PB
0.527798663	Only oral AB
0.336765068	Oral AB and Local AB+PB
0.61261063	Only local AB
0.603473837	Only local AB
0.620889847	Only local AB
0.217817808	Only local AB
0.641141465	Only local AB
0.298416299	Only local PB
0.802510214	Only local PB
0.211821825	Only oral PB

tx2_lab	A
Local AB and Oral PB	Only local AB (5-nitroimidazole)
Only local AB	Local AB (5-nitroimidazole) + PB
Only local AB	Local AB (clyndamicin) + PB
Only local AB	Only oral AB (5-nitroimidazole)
Only local AB	Only oral AB (5-nitroimidazole)
Only local AB	Only oral AB (clyndamicin)
Only local PB	Only local AB (5-nitroimidazole)
Only local PB	Only local AB (5-nitroimidazole)
Only local PB	Oral AB (clyndamicin) and Local AB (5-nitroimidazol
Only oral AB	Only local AB (clyndamicin)
Only oral AB	Oral AB(5-nitroimidazole) + PB
Only oral AB	Oral AB(5-nitroimidazole) + PB
Oral AB and Local PB	Only oral AB (5-nitroimidazole)
Oral AB and Local AB	Oral AB (clyndamicin) and Local AB (5-nitroimidazol
placebo	Only local AB (5-nitroimidazole)
placebo	Only local AB (5-nitroimidazole)
placebo	Only local AB (5-nitroimidazole)
placebo	Only local AB (clyndamicin)
placebo	Only local AB (clyndamicin)
placebo	Only local PB
placebo	Only local PB
placebo	Only oral PB

B
Local AB (5-nitroimidazole) and Oral PB
Only local AB (5-nitroimidazole)
Only local AB (clyndamicin)
Only local AB (5-nitroimidazole)
Only local AB (5-nitroimidazole)
Only local AB (clyndamicin)
Only local PB
Only local PB
Only local PB
Only oral AB (5-nitroimidazole)
Only oral AB (5-nitroimidazole)
Only oral AB (5-nitroimidazole)
Oral AB (5-nitroimidazole) and Local PB
Oral AB (clyndamicin) and Local AB (5-nitroimidazole)
placebo
placebo
placebo
placebo
placebo
placebo
placebo
placebo

tx1_lab
1atb(oxofloxacino200+ornidazol500)-local-1vezdia/5dias-2(co-kimaxazol)local-1v3d
1atb(tinidaz2000)-local-1v/1d/2atb(metro)-local-1v/2dias/3pro(la+lrh)-local-1v/35d
1atb(clindamicina100)-oral-1v/7d/2pro(lg+lrh)-local-1v-10d
Oral metro
metro oral
Oral clnda
1atb(metro37.5)-local2v/5d
intravag metro
pro(la+lr)-local-1v10dias
Local Clionda
1atb(metro1000)-oral1v/7d/2pro(lrh+lr)-oral2v/30dias
1atb(tinidaz2000)-oral1v/1d/2pro(rrh-lr)-oral1v/2dias
atb(metro500)-oral-2vezdia/7dias
pro(la+lr)-local-1v10dias
atb(metro100)-local-1vezdia/14dias
atb(metro250)-local-1vezdia/14dias
atb(metro500)-local-1vezdia/14dias
atb(clindamicina100)-local-1v/7d
atb(clinda100)-local-1v/7d
3d-prO(lf-lrh-lga)-local
pro(L.bre-saliv-plant)-local-1vezdia/7dias
pro(L.rh+L.r)-oral-2dias/42dias

tx2_lab
1atb(oxofloxacino200+ornidazol500)-local-1vezdia/5dias-2(co-kimaxazol)local-1v3d/3pro-B. coagulans Unique IS-2
Vaginal metro
metro vaginal
Vaginal clnda
pro(lr+lrh)-local-1v5dias
intravag L. delbrueckii
1atb(clind600)-oral-1v5dias/2atb(metro1000)local1v5d
oral metro
1atb(tinidaz2000)-oral1v
1atb(metro500)-oral-2vezdia/7dias-2pro(lrh)-local-1v/8d
1atb(clind600)-oral-1v5dias/2atb(metro1000)local1v5d/3pro(la+lrh)-local-1v/35dias
